# How to Work with the Microscope

**Published:** 1860-11

**Authors:** 


ART. X.—*Illustrations How to Work with the Microscope.* By LIONEL BEALE, M.D., F.R.S. London, 1859.

THE illustrations in this little brochure comprise upwards of one hundred and fifty separate figures, executed in the highest style of the art, and well calculated to aid the student in the use of the microscope, at present so much in vogue in the investigation of the morbid conditions of the system. The author, Professor Beale, of King's College, London, is most favorably known for his zeal in minute anatomical researches, and his larger work, entitled the "Microscope in Medicine," has given him an enviable reputation on both sides of the Atlantic.

